# The gut virome of healthy children during the first year of life is diverse and dynamic

**DOI:** 10.1371/journal.pone.0240958

**Published:** 2021-04-14

**Authors:** Blanca Taboada, Patricia Morán, Angélica Serrano-Vázquez, Pavel Iša, Liliana Rojas-Velázquez, Horacio Pérez-Juárez, Susana López, Javier Torres, Cecilia Ximenez, Carlos F. Arias

**Affiliations:** 1 Instituto de Biotecnología, Universidad Nacional Autónoma de México, Cuernavaca, Morelos, México; 2 Unidad de Investigación en Medicina Experimental, Facultad de Medicina, Universidad Nacional Autónoma de México, Ciudad de México, México; 3 Unidad de Investigación Médica en Enfermedades Infecciosas y Parasitarias, Hospital Pediatría, Centro Médico Nacional Siglo XXI, Instituto Mexicano del Seguro Social, Ciudad de México, México; Arizona State University, UNITED STATES

## Abstract

In this work, we determined the diversity and dynamics of the gut virome of infants during the first year of life. Fecal samples were collected monthly, from birth to one year of age, from three healthy children living in a semi-rural village in Mexico. Most of the viral reads were classified into six families of bacteriophages including five dsDNA virus families of the order *Caudovirales*, with *Siphoviridae* and *Podoviridae* being the most abundant. Eukaryotic viruses were detected as early as two weeks after birth and remained present all along the first year of life. Thirty-four different eukaryotic virus families were found, where eight of these families accounted for 98% of all eukaryotic viral reads: *Anelloviridae*, *Astroviridae*, *Caliciviridae*, *Genomoviridae*, *Parvoviridae*, *Picornaviridae*, *Reoviridae* and the plant-infecting viruses of the *Virgaviridae* family. Some viruses in these families are known human pathogens, and it is surprising that they were found during the first year of life in infants without gastrointestinal symptoms. The eukaryotic virus species richness found in this work was higher than that observed in previous studies; on average between 7 and 24 virus species were identified per sample. The richness and abundance of the eukaryotic virome significantly increased during the second semester of life, probably because of an increased environmental exposure of infants with age. Our findings suggest an early and permanent contact of infants with a diverse array of bacteriophages and eukaryotic viruses, whose composition changes over time. The bacteriophages and eukaryotic viruses found in these children could represent a metastable virome, whose potential influence on the development of the infant’s immune system or on the health of the infants later in life, remains to be investigated.

## Introduction

Humans are in constant close contact with a rich variety of bacteria, archaea, fungi, protists and viruses, which all together reside on, or within the human body, forming an ecosystem denominated microbiota. Distinct body sites are characterized by distinct microbial communities, with the gastrointestinal tract being the most populated site, playing an important role in human health and development. The bacterial component of the microbiota is the best characterized, having many functions in host physiology like metabolic processes, development and maturation of the immune system [1, 2]. Bacterial colonization begins during birth and continues to change and evolve throughout life and its composition can be influenced by factors as birth mode, gestational age, antibiotic usage, diet, geographical location, lifestyle and age [3, 4]. Modifications in the microbiota composition can lead to several diseases, including obesity, diabetes, or cardiovascular diseases, among others.

Contrary to bacterial composition, the virus assembly (known as virome) in the gut is much less understood. It has been observed that bacteriophages are the predominant viruses, where *Siphoviridae*, *Inoviridae*, *Myoviridae*, *Podoviridae* and *Microviridae* represent the most common families [5–7]. Eukaryotic viruses have also been commonly found in the gut of healthy individuals, with animal viruses of the *Anelloviridae*, *Picobirnaviridae*, and *Circoviridae* families being the most frequently reported [8–11], although viruses from the *Adenoviridae*, *Astroviridae*, *Caliciviridae*, *Parvoviridae*, *Picornaviridae*, and *Polyomaviridae* families have also been described [8, 9]. In addition, the presence of different families of plant viruses, including *Alphaflexiviridae*, *Tombusviridae*, *Nanoviridae*, *Virgaviridae* and *Geminiviridae*, have also been commonly reported.

Much less is known about the gut virome composition of healthy children during first years of life. Bacteriophages show high diversity and dynamic in the first months of life, decreasing over time and becoming stable until 2 years old. A number of phages have been identified to be shared between mothers and infants, suggesting their transmission through the mothers’ milk [12, 13] or through the placenta [14]; although the most abundant phages detected in the infants were not found in their mothers [15] or the milk or the babies formula [12]. In infant twins, phages are more similar between them than in unrelated children [15–17]. Moreover, other factors as birth delivery mode and feeding type have been identified to affect the diversity of bacteriophages [12, 15, 18, 19]. Regarding eukaryotic viruses in healthy infants [13, 15, 16, 18–22], they have been identified sporadically, being less frequent in the first xx months of life and increasing in frequency with age. Viruses known to cause illness, from *Adenoviridae*, *Anelloviridae*, *Astroviridae*, *Parvoviridae*, *Picornaviridae*, *Picobirnaviridae* and *Reoviridae* families, have been commonly identified in infant stool samples, even in the absence of clinical symptoms [13, 16, 18, 20, 22–24].

In this work, the viral composition of the gastrointestinal tract of three healthy infants from a semi-rural town in Mexico was characterized in fecal samples collected monthly during their first year of life. The presence of eukaryotic viruses was detected as early as two weeks after birth and represented a diverse metastable and dynamic virome along the year of study formed by a rich and abundant mixture of viruses from plant and animal hosts.

## Materials and methods

### Population studied and sample collection

This study was carried out in the semi-rural community of Xoxocotla, in the state of Morelos, 130 km south of Mexico City. Healthy women who arrived for routine control at the pregnancy clinic of the Xoxocotla Health Center, during the last trimester of pregnancy, were invited to participate in the study. Written informed consent was obtained from all mothers after providing them with detailed information about the study and its characteristics. The protocol and the consent letter were approved by the Scientific and Ethics Committee of the Medical School of the National University of Mexico as well as by the Ministry of Health of the State of Morelos. Three mother-infant pairs were included in this study; the infants were healthy, full-term products, without any congenital condition and with normal weight at birth. A single fecal sample was obtained at the end of the last trimester of pregnancy from each mother. The infants were followed monthly during their first year of life between March 2015 and June 2016. The stool samples were collected immediately after defecation by the mothers, previously trained and under the supervision of a responsible nurse. Sterile plastic containers with screw caps were used to collect stool samples from the diapers; samples were not exposed to antiseptics or disinfectants. The samples were kept at 4°C and transported to the village laboratory where they were kept at -20°C for less than 1 week; The samples were transported to the Institute of Biotechnology where they were stored at -70°C, until use.

### Nucleic acid isolation and sequencing

Nucleic acids were extracted from the stool samples as described before [25]. Briefly, 10% stool homogenates were prepared in phosphate-buffered saline (PBS); the, chloroform (10%) and 100 mg of 150 to 212 μm glass beads (Sigma, USA) were added in final volume of 1 ml and processed in a bead beater (Biospec Products, USA). The samples were centrifuged at 2000 x g to remove large debris, and the recovered supernatants filtered through Spin-X 0.45 μm pore filters (Costar, NY). A volume of 400 μl of filtered samples was treated with Turbo DNAse (4 U) (Ambion, USA) and RNAse (0.3 U) (Sigma, USA) for 30 min at 37°C. Nucleic acids were extracted using the PureLink viral RNA/DNA extraction kit according to the manufacturer’s instructions (Invitrogen, USA), and eluted in nuclease-free water, aliquoted, and stored at -70°C until further use. Nucleic acids were random amplified with SuperScript III reverse transcriptase (Invitrogen, USA) with primer A (5’–GTTTCCCAGTAGGTCTCN_9_-3’). The cDNA was generated by two consecutive rounds of synthesis with Sequenase 2.0 (USB, USA). The synthesized cDNA was then amplified with Phusion High fidelity polymerase (Finnzymes) using primer B (5’-GTTTCCCAGTAGGTCTC-3’) and 10 additional cycles of the program: 30 sec at 94°C, 1 min at 50°C and 1 min at 72°C. Then, the DNA was purified using ZYMO DNA Clean & Concentration-5 kit. Sequencing libraries were prepared using Nextera XT DNA library preparation kit (Illumina); samples were uniquely tagged, pooled and then deep sequenced on the Illumina NextSeq500 system, generating paired-end reads of 75 bases. The base calling was performed by Illumina Real Time Analysis (RTA) v1.18.54 software and the demultiplexing of reads by bcl2fastq v2.15.0.4.

### Metagenomic data analysis

A viral metagenomics pipeline (S1 Fig), which includes quality controls, filtering and taxonomic annotation was applied as previously described [26]. Briefly, the process was: i) Quality control. Adapters, low quality bases from 5’ and 3’ ends, low complexity reads or shorter than 40 bases were removed [27] and exact duplicates reads were excluded [28]. ii) Filtering. Ribosomal RNA and human genome reads were filtered using ribosomal Silva database (DB) [29] and human genome from GenBank [30]. The remaining sequences were considered valid reads. iii) Taxonomic classification. Valid reads were mapped to viral-bacteria-fungi reference DBs obtained from nt of NCBI [31], using SMALT [30], and mapped reads were assembled using IDBA-UD software [32]. Assembled contigs and singlets reads were compared against nt DB using BLASTn [33] to remove false positives. Then, the reads that did not map using the nucleotide alignment were assembled and contigs greater than 200 bases were compared to all protein sequences of nr DBusing BLASTx. Then, the software MEGAN 5.10.6 [34] was used to assign both individual reads and contigs (with magnitude) to the most appropriate taxonomic level. Single reads plus the total number of reads contained in all contigs assigned to all taxa, with at least 5 of magnitude, were extracted from MEGAN to generate count matrix. Differences in the sequencing depth of the samples were corrected by dividing the number of bacteria and virus reads by each sample valid reads and normalized to 5 million per sample. These values were used to generate final abundance tables at different taxonomic levels. Iv) Functional Analysis. All contigs obtained in the above process were used to predict and extract protein-coding ORFs greater than 200b by using Prodigal in ‘normal’ mode. Then, they were compared to viruses and bacteria proteins of nr DB, in order to functional annotate them.

### Statistical analysis

The taxa abundance differences were analyzed using Trimmed Mean of M-value (TMM) method [35]. Unless otherwise indicated, statistical analyses were conducted in R-3.5.3 statistical environment [36], using the Vegan package [37]. To assess the alpha diversity, we calculated richness as the expected number of species, Shannon diversity (H) index and Pielou’s using the final abundance tables described in the previous section (S1 Fig). For beta diversity, Bray–Curtis distance metric was used [38]. The distances were used as input for the Nonmetric Multidimensional Scaling (NMDS) ordination method. For comparison of groups, samples were divided into cases and controls and in order to associate them with metadata factors, a nonparametric multivariate permutation test (PERMANOVA) analysis was done using the Adonis function with 999 permutations, and Mann-Whitney’ test for measures [39]; homogeneity variances between groups were verified in all comparisons. Finally, the differential abundance of taxonomic units was carried out with the EdgeR package (version 3.24.3) in R (version 3.5.3), as described in Loraine et al. [40]. Common and tag-wise methods were used to estimate the biological coefficient of variation and “exact” test used to perform hypothesis testing and false-discovery rates to adjusted p-values. All statistics were considered significant if p < 0.05.

## Results

### Infant´s cohort and sample analysis

Fecal samples from three apparently healthy infants, with no disease symptoms during the study, were collected monthly, starting two weeks after birth and until 12 months of age, obtaining 11 samples from infant 2, and 12 samples from infants 4 and 5. Mother samples were taken at a single time point around the eighth month of pregnancy. The characteristics of mothers and infants are described in S1 Table. Two infants were born via cesarean section (infant 4, male; infant 5, female), while infant 2 (male) was born via vaginal delivery. The three infants were breastfed all along the year of study, although they received supplemental formula after their first three months of life. Only the mother of infant 2 was exposed to antibiotics before sample collection, but none of the infants received antibiotics throughout the study, but they received doses of BCG, Hepatitis B, Influenza, Pentavalent, Pneumococca and Rotavirus vaccines.

Total nucleic acids were extracted from 38 fecal samples (35 from infants and 3 from mothers) to detect both DNA and RNA viruses and sequenced using the paired-end 2×75 bp Illumina HiSeq 500 system (Illumina, Inc). Initially, 421.78 million paired-end reads were obtained for all libraries, with a mean of 12.05 ± 3.23 millions per sample (S2 Table). After quality control, duplication removal and filtering (human and ribosomal) processes, 90.84 million valid reads remained (mean = 2.39 millions, ranging from 0.68 to 3.58).

### Viral taxonomy composition

**Fig 1:**
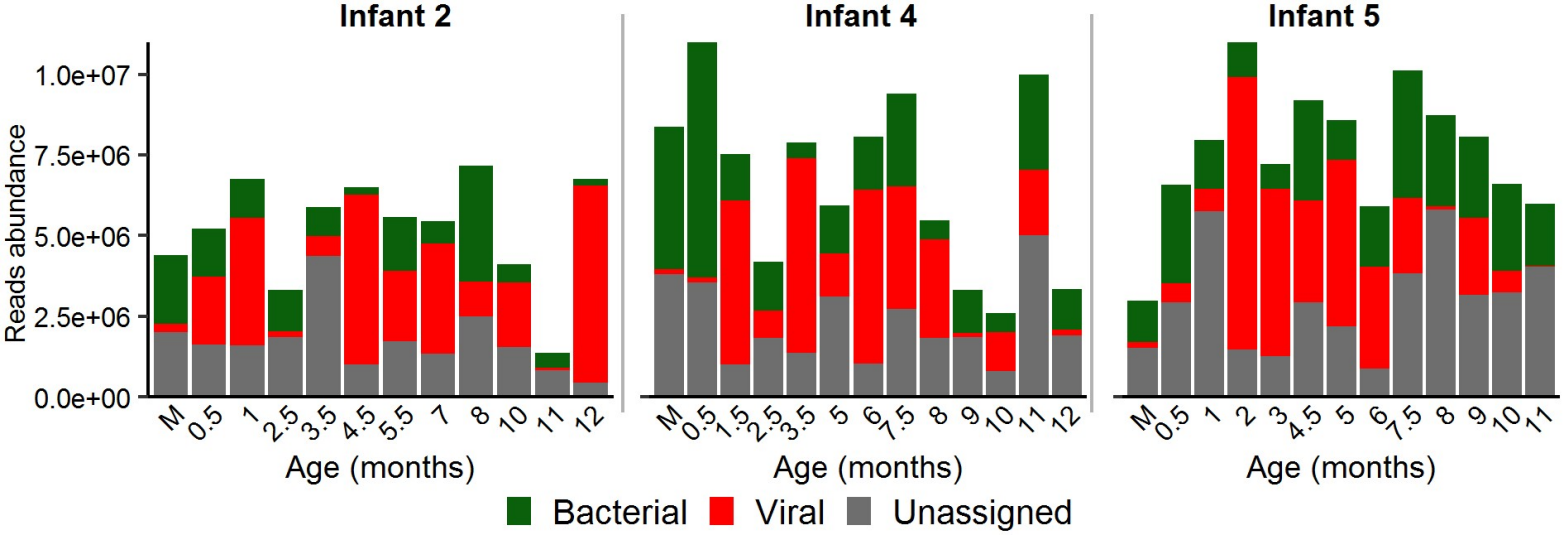
Abundance of bacterial and viral sequence reads in each sample. Samples from infants are indicated according to their age in months and from mothers as M.

On average, it was found that 36.1% (±27.6% s.d.) and 26.9% (±14.3% s.d.) of the valid reads in the infant samples had homology to at least one viral or bacterial reference sequence, respectively (Fig 1). In contrast, in mothers only 4.2% (±2.4% s.d.) of valid reads were identified as viral, whereas bacterial reads were as high as 47.9% (±5.0% s.d.). Similar to previous viral metagenomics studies, 37.8% (±17.8% s.d.) of valid reads per sample showed no significant similarity to any known sequence of GenBank DB [17, 41]. Reads mean percentages were calculated for each sample first and then, the global mean for all samples was estimated.

### Eukaryotic viruses

In infants, 15.5% of the assigned viral reads correspond to eukaryotic viruses, which can be classified within 33 families (S2 Fig), about half of them infect vertebrates (42.4%), followed by those that infect plants (21.2%), plants/fungi (12.2%), invertebrates (12.2%), amoebae (6.0%), algae (3.0%) and fungi (3.0%). It is important to point out that nine of the viral families identified accounted for 97% of all reads: i) *Virgaviridae*, the most abundant, was identified in all samples (35/35), representing an average of 26.6% of all eukaryotic viral reads. ii) *Anelloviridae* and *Picornaviridae* represented on average 24.8% and 18.7% of all eukaryotic viral reads, respectively, and were found in up to 80% of the samples. iii) *Caliciviridae*, *Parvoviridae* and *Reoviridae* were less abundant, on average between 5.8%-8.8%, and less prevalent, been found in around 60–71% of samples. Iv) *Astroviridae* and *Genomoviridae* were identified in between 43–49% of the samples, while *Circoviridae* in only 17%. As expected, the most abundant and prevalent viral families found have humans as hosts, except for the family *Virgaviridae*, whose natural hosts are plants. On the other hand, among the viral families identified in a single sample or in less than 10% of the samples, only 15% infect invertebrates. In the samples from the three mothers, 18.6% of the viral reads were eukaryotic, classified into 16 families, and the rest were bacteriophages, with *Virgaviridae* (90.6%) and *Phycodnaviridae* (8.3%) as the dominant virus families.

At the genus level, 54 viral genera were identified in the infant samples and 18 were present in the samples obtained from their mothers (S3 Fig). *Tobamovirus* of the *Virgaviridae* family was the most prevalent genus, being found in all mother and infant samples, and was also the most abundant in infants and mothers, representing 28.5% and 91% of the eukaryotic viral reads per sample, respectively. A detailed characterization of the plant viruses found in the infants’ gut was recently reported by our group [42].

**Fig 2:**
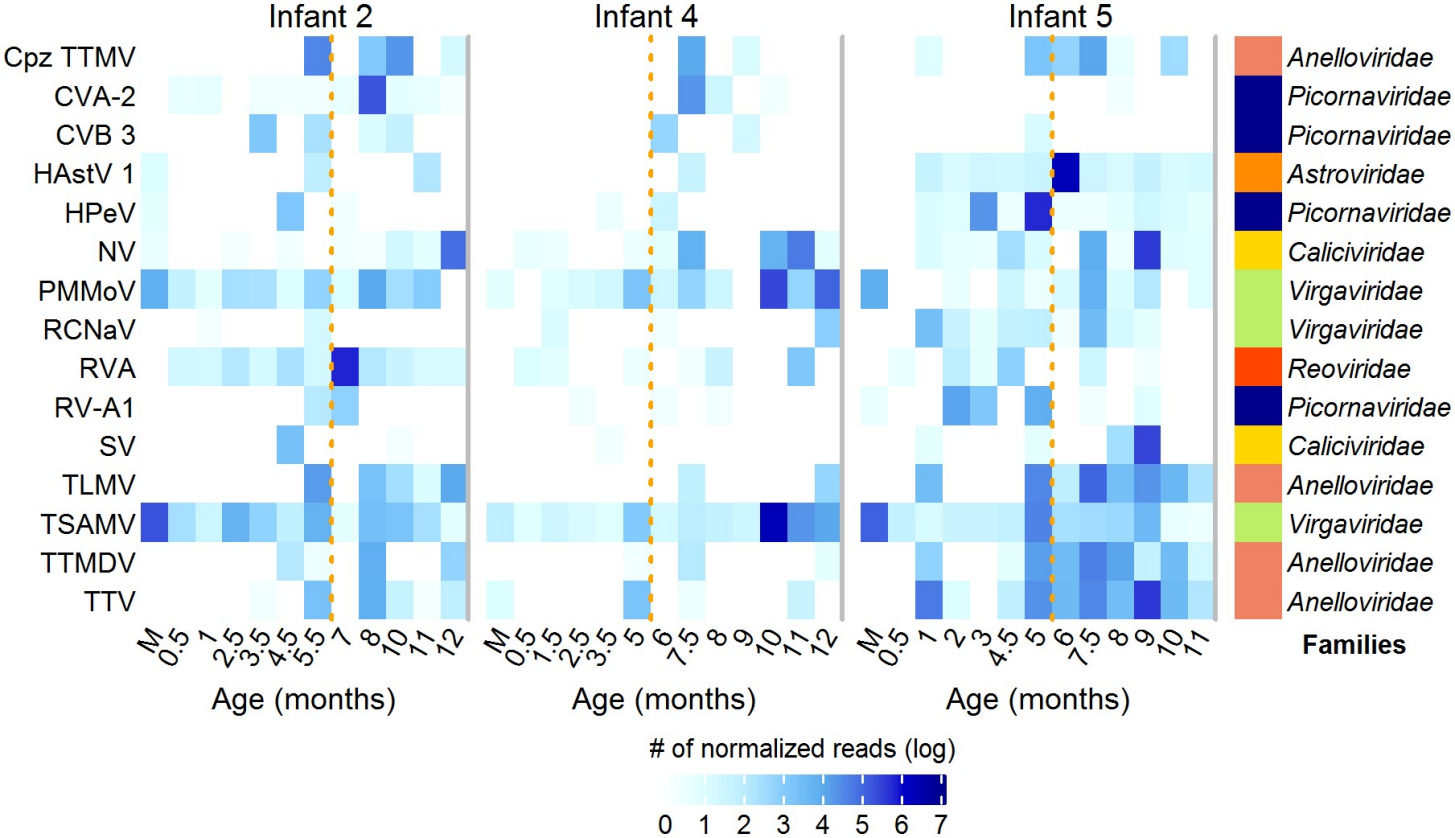
Normalized read abundance of the most abundant and prevalent eukaryotic viral species in infants during the first year of life and their mothers. Samples from infants are indicated according to their age in months and from mothers as M. The family to which each virus species belongs is indicated on the right side. *Cpz TTMV, chimpanzee torque teno mini virus; CVA-2, enterovirus A; CVB 3, enterovirus B; HAstV 1, human astrovirus 1; HPeV, parechovirus A; NV, Norwalk virus; PMMoV, pepper mild mottle virus; RCNaV, rattail cactus necrosis-associated virus; RVA, rotavirus A; RV-A1, rhinovirus A; SV, Sapporo virus; TLMV, TTV-like mini virus; TSAMV, tropical soda apple mosaic virus; TTV, torque teno virus and TTMDV, torque teno midi virus. The discontinuous yellow lines divide the year into semesters.

In contrast to previous studies, we were able to classify reads at lower taxonomic levels, identifying 124 eukaryotic viral species in infants and 30 in mothers (S3 Table). On average, 15 (±9 s.d.) viral species were identified per infant sample and 12 (±1) in each mother´s sample. Most species were identified sporadically, with 70% of them being observed in only 10% of infant´s samples. However, some species were frequently found in samples throughout the year. Fig 2 shows the most common and abundant species across all samples. Interestingly, tropical soda apple mosaic virus (TSAMV) and pepper mild mottle virus (PMMoV), from the plant infecting *Virgaviridae* family, were the most prevalent and abundant species in both, infant and mother samples. TSAMV was found in all samples, representing on average 15.9% of the eukaryotic viral reads per infant sample (range 0.001–89.4%) and 62.9% of reads in the mothers´ samples (range 0.7–95.9%). Whereas PMMoV represented, in average, 10.3% of the viral reads in the infant samples and 3.5% in the mothers’ samples; this virus was detected in 80% and 100% of infant and mother samples, respectively. Other abundant virus species found in more than half of the infant´s samples were Norwalk virus (NV), rotavirus A (RVA) and torque teno virus (TTV); there were also other 8 viral species identified in at least 30% of the samples and others that were identified sporadically (S3 Table).

Interestingly, complete or partial (>90% of coverage) genomes were obtained from several viral species, by assembling them individually, such as, human bocavirus (4 genomes), enterovirus (3), human astrovirus (2), human rotavirus (3), norovirus (6), parechovirus (2), pepper mild mottle virus (7), rattail cactus necrosis associated virus (1), tomato mosaic virus (1), tropical soda apple mosaic virus (9) and sapovirus (2).

### Bacteriophages

As previously observed [13, 16, 17, 43–45], the vast majority of viral reads identified were classified into six different families of bacteriophages, with 84.5% of abundance in infants and 81.4% in mothers (S4 Fig). In infants, five dsDNA families of the order *Caudovirales* were the most abundant: *Siphoviridae* (long, non-contractile tailed-phages, temperate, with some lytic members), with an average of read abundance of 51.5% per sample; *Podoviridae* (short, contractile tailed-phages, lytic, with some temperate members) with 20.2%; *Myoviridae* (long, contractile tailed-phages, strictly lytic) with 12.7%; the provisional family crAss-like (contractile tailed-phages, strictly lytic, with podovirus-like morphology) with 10.4%; and *Ackermannviridae*, the new bacteriophage family proposed in 2017, with 2.9%. The ssDNA family *Microviridae* (lytic, with some identified as prophages) represented, on average, 2.3% of children´s bacteriophage reads. Interestingly, in mothers, the crAss-like bacteriophages predominated in the gut, with an average of 79% of the total phage reads.

Regarding phage genera, 97 were identified in infants and 31 in mothers (S4 Table), with the genus *Pis4avirus* from the *Siphoviridae* family and the genus *G7cvirus* from the *Podoviridae* family being the most common, since they were identified in all infant samples. Also, 8 other frequent and abundant genera were identified in more than 75% of samples, while more than 31 in a single sample or in less than 3 in the infants.

**Fig 3:**
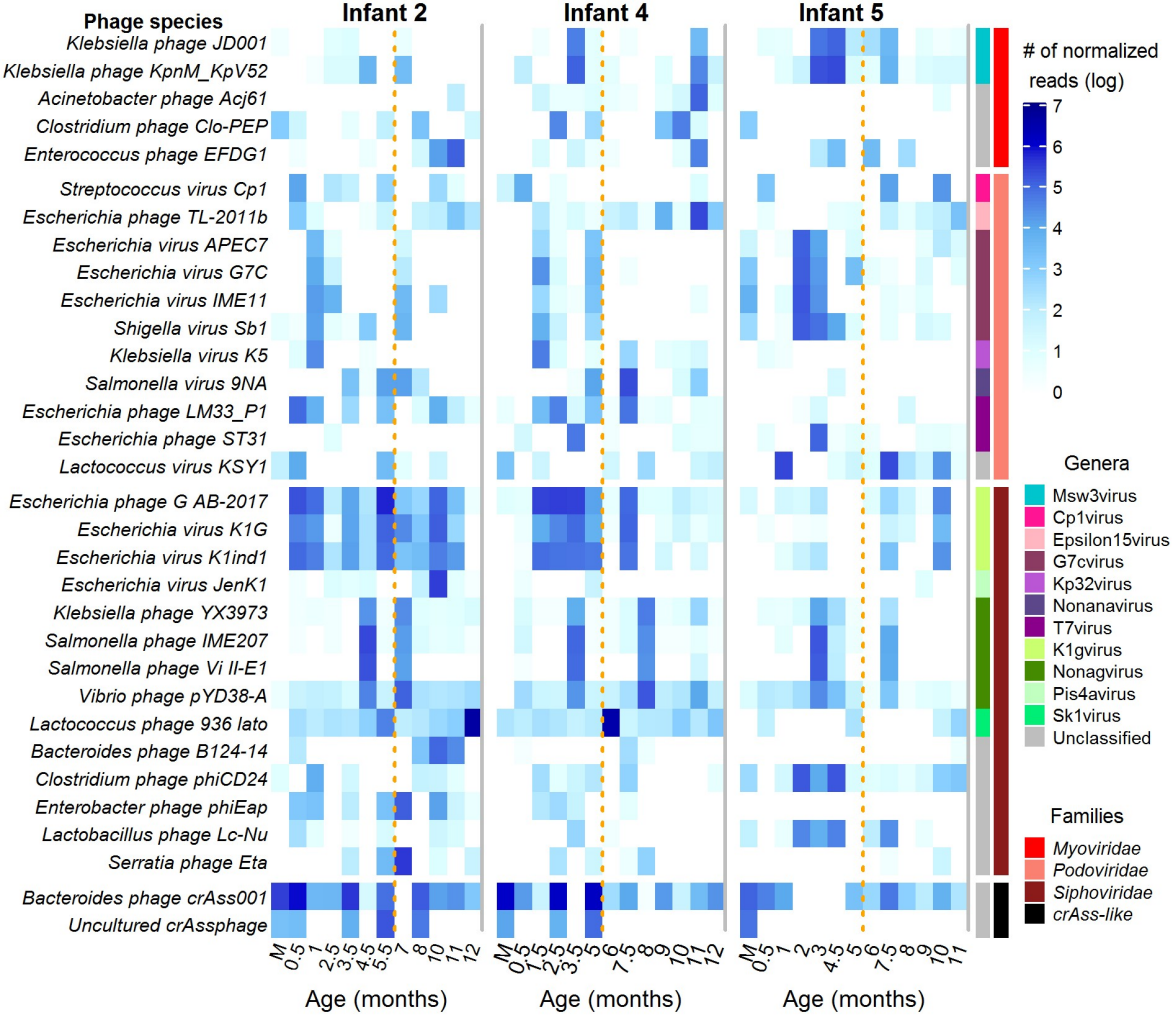
Normalized read abundance of the most abundant and prevalent phage species in infants during the first year of life and their mothers. Samples from infants are indicated according to their age in months and from mothers as M. The family for each phage species is indicated on the right side. The name of the phage species is listed on the left side.

In contrast with previous reports that have shown that a stable community of bacteriophages exist in adults over a long period of time [5, 6], we observed a dynamic and unstable community of phages in infants during their first year of life (S5 Table), in agreement with previous studies in early childhood [16, 46]. The majority were identified in a single sample with 48% of the phages (266 species) found in only one or two samples. However, we found 82 species shared in one third of the infant samples, 29 species in 50%, and 7 species in more than 80% of the samples. This more stable phageome included phages of high abundance, e.g., the 7 most frequently species also accounted for 33% of the total read abundance (Fig 3). On average, infants harbored 79 species per sample (range 36–194), 18 from the *Myoviridae* and *Podoviridae families*, 40 from the *Siphoviridae* and 1 from the *Ackermannviridae*, crAss-like and *Microviridae*.

The most abundant and frequent phage species in both infants and mothers has around 75% of homology to the Bacteroides phage crAss001 from the crAss(cross Assembly)-like family (Fig 3). This phage has been recently reported to be the most abundant and prevalent type of crAssphage in the adult human gut, and to infect bacteria of the order *Bacteroidales* [47]. We found it in all mothers’ samples and in 87% of the infants’ samples, with an average abundance of 71.7% and 13.7% of total phage reads per sample, respectively. Although this group of phages are diverse, the crAssphages identified in this work had homology only to crAssphage Azobacteroides phage, Bacteroides phage, Cellulophaga phage, IAS virus and one uncultured crAssphage. Other common and abundant bacteriophages in infants showed identity to different species of *Escherichia*, *Lactococcus*, *Salmonella*, *Streptococcus*, *Klebsiella*, *Staphylococcus*, *Clostridium* and *Enterococcus* phages, with more than 50% of prevalence (Fig 3).

**Fig 4:**
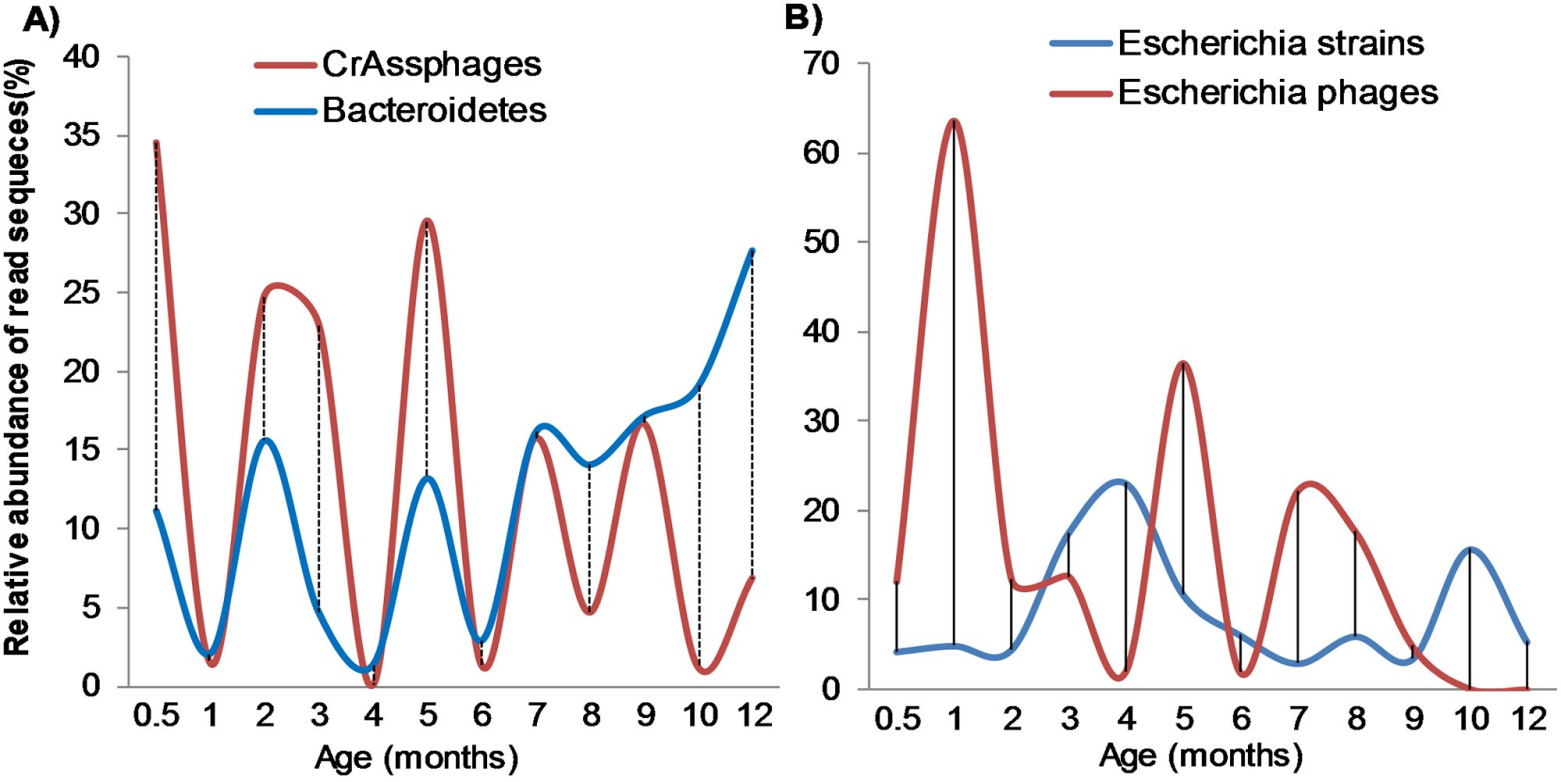
Average abundance of reads of the two most represented phages and their bacteria hosts in the infants, all year long. A) Lytic phage CrAssPhges and their Bacteroidetes hosts. B) Lytic Escherichia phages and their Escherichia bacteria hosts.

Even though the samples were filtered through 0.45 filters before nucleic acid extraction, to reduce bacterial contamination, about 49.4% of the total reads were classified as bacterial. Not surprisingly, the bacterial hosts of the most abundant and persistent phages in infants were identified, and they followed during the period of study two relationship patterns: a) Positive correlation, where bacteria-phages abundances move in tandem, b) Negative correlation, where bacteria increase as the viruses decrease, and vice versa. For example, at 15 days of age, the crAssphages were more abundant (on average 35%) than the Bacteroidetes (12%), a trend that was positively correlated during the first semester, in a ratio that started to change during the second semester of life, becoming the inverse; thus, by month 10 the Bacteroidetes phages were more abundant than the CrAssphages (Fig 4A). An additional example are the *E*. coli virulent phages in the *Myoviridae* and *Podoviridae* families that were abundantly identified in some samples, persisting for prolonged periods of time (Fig 4B). These phages infect *Escherichia* spp and *Klebsiella* spp, showing negative correlation with them along the year. To understand more phage-bacteria relationships in the gut of infants, we also analyzed viral contigs, using blastp (S1 Fig), to determine whether there were proteins used in the lysogenic cycle of phages (integrases and proteases) or proteins used in the lytic cycle (holin, portal and endolysins) [48]. In total, 29,276 viral contigs greater than 300 bases were identified, and we assigned function to 61% (17,861) of them. Approximately, 8.8% of annotated viral contigs were lysogenic proteins and 4.3% lytic ones.

### Alpha and beta diversity

**Fig 5:**
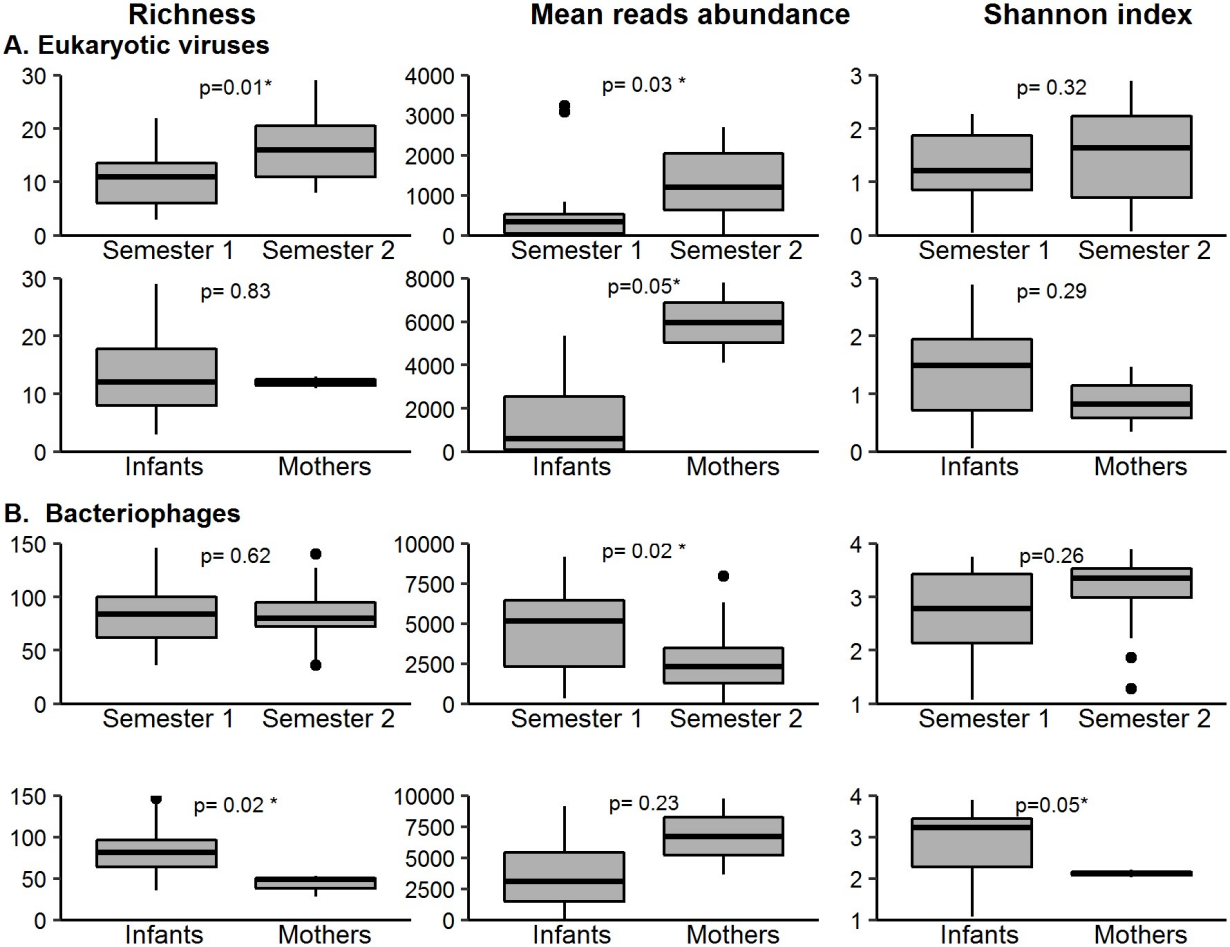
Bar plots representing viral diversity metrics as Chao richness index, mean abundance per species and Shannon diversity index. The metrics of the first vs. the second semester of infants, as well as the metrics of mothers as compared to those of infants, are compared. A) Eukaryotic viral diversity metrics. B) Bacteriophage diversity metrics.

Diversity estimations were performed using the normalized matrixes of viral read and contig counts at the species level, and their association with factors of metadata (Fig 5). In infants, the Shannon alpha diversity of eukaryotic viruses was 1.4 ± 0.7 (Fig 5A) and of bacteriophages 3.2 ± 0.8 (Fig 4B), while in mothers these values were 0.8 ± 0.7 and 1.6 ± 0.8, respectively. In the diversity analysis, we found: i) the second semester of life of the infants was significantly richer (p = 0.01) and more abundant (p = 0.03) in eukaryotic viruses than the first semester (Fig 5A); ii) in contrast to this, phages were more abundant in the first semester (Fig 5B, p = 0.02), iii) the diversity of bacteriophages was greater in infant 5 when compared to the other two infants, while in the eukaryotic virome only the richness was increased in this infant, iv) bacteriophages were significantly richer (p = 0.02) and more diverse (p = 0.05) in infants as compared to mothers (Fig 5B), as has been found in previous studies that have compared infants and adults [13, 16, 17, 43–45]; v) the eukaryotic virome, contrary to the phageome, was more abundant in mothers than in infants (Fig 5A, p = 0.05).

**Fig 6:**
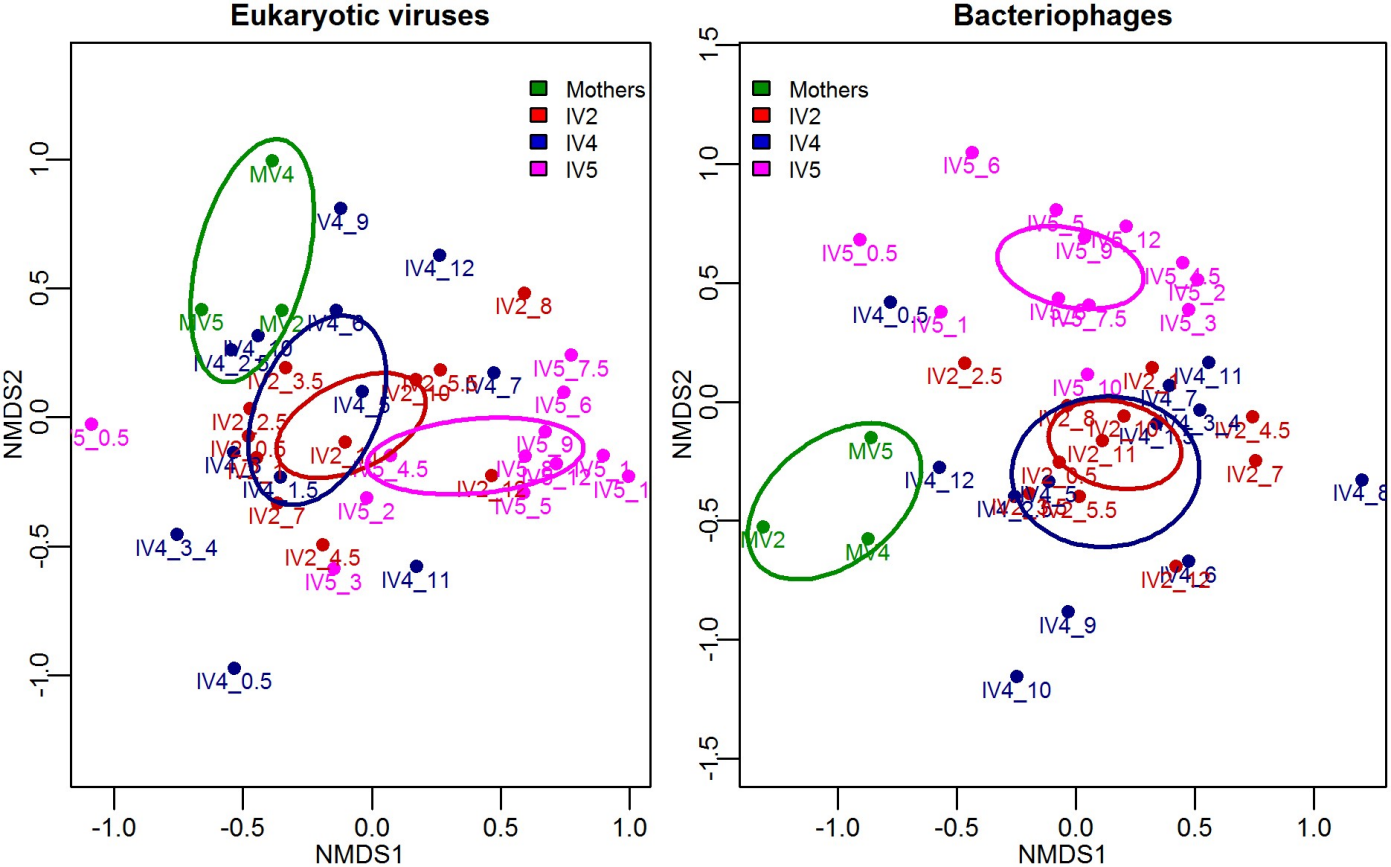
Multidimensional Scaling (MDS) analysis of eukaryotic viruses and bacteriophages at the species level. Bray-Curtis dissimilarity distances from normalized counts were used. Each point corresponds to a sample, and ellipses represent the standard errors of the centroids of the types of samples (mothers, infant 2, infant 4 and infant 5). Ellipses were calculated using the Ordiellipse function of the R package ‘vegan’ [37], at 95% confidence.

The beta diversity of the eukaryotic viruses and of bacteriophages was calculated among mothers and infants, to individually compare them and to discern if there were patterns in association with metadata. Fig 6 illustrates the result of a Multidimensional Scaling (MDS) analysis using this beta diversity. Statistical analysis showed agreement with alpha diversity, obtaining significant difference between phage communities of the samples from mothers and infants (PERMANOVA using Adonis p = 0.002); however, such difference was not seen among eukaryotic viruses (p = 0.1). In both types of viruses, there was a difference between infant 5, who is a girl, and the other two infants (2 and 4) (p = 0.003), who are boys. Regarding age, there was a difference between eukaryotic viruses in the first and second semester of life (p = 0.01), but such difference was not observed when the phages were analyzed (p = 0.09).

## Discussion

It is of upmost importance to understand the way the enteric virome develops during infancy and what impact on the development of the gastrointestinal tract and the human health it may have. Few studies have described the gut virome of infants during the first year of life [12, 13, 15–19, 21, 43–45, 49], with even fewer studies carried out in healthy children, in the community [13, 16–18, 21]; only four of these studies have been longitudinal [16, 19, 20, 22]. In this work, we characterized the monthly gastrointestinal virome, prokaryotic and eukaryotic, of three healthy infants during their first year of life.

Regarding eukaryotic viruses, 33 families were identified in infants, but only nine were frequently found and made up to 97% of all eukaryotic viral reads identified. Aside for plant-infecting viruses in the *Virgaviridae* family, the most abundant and frequently found were viruses belonging to the families *Anelloviridae*, *Astroviridae*, *Caliciviridae*, *Genomoviridae*, *Parvoviridae*, *Picornaviridae* and *Reoviridae*. Some viruses in these families are common human pathogens, especially in children; it is thus surprising that they were commonly found in the absence of gastrointestinal symptoms all along the year of the study. The gut mucosa of infants is under a process of maturation and receptors to pathogens may still be absent; although other immunological or nutritional factors may also be involved.

In our study, members of the *Anelloviridae* family were highly abundant, containing different species of torque teno mini virus and unclassified species in up to 80% of the samples. In previous studies, viruses from this family have been identified as the most abundant and frequent in healthy children [14, 15, 17–19, 22–24, 50, 51], being more abundant during the first year of life [16, 17, 22, 24], after which the abundances decrease. Their presence has been associated with a reduced host immune status; a higher abundance have been reported in patient with lung transplantation [52, 53], AIDs [54], pulmonary diseases [55, 56], cancer [57], among others; although their role in the pathogenesis of these diseases remains unclear [58]. Our results showed that anelloviruses were significantly more abundant in the second semester of life compared with the first one (P-value 0.01, S6 Table), specially TTV like mini virus and torque teno virus species. These results agree with Lim et. al. [16] and suggest that infants come more in contact with these viruses a few months after birth, from an unknown source. Viruses in the *Picornaviridae* family have also been frequently found in healthy children [14, 19–23, 50], even in Tan et. al [22] picornaviruses represented the vast majority of reads (93.6%). In our study, this family was identified in 80% of the infant samples, with parechovirus A and enterovirus A being the most common species. The duration of parechovirus secretion in the stool of healthy infants has been reported to last between 41 and 93 days [59]; in this regard, we also detected parechoviruses in infant feces during two consecutive months, followed by periods of null or undetectable levels.

Viruses in the *Caliciviridae* family have been found at a low frequency (<7%) in healthy infants in metropolitan areas of the USA [16, 19], South Africa [21] and Bangladesh [22], while they were absent in an urban city but frequent (45%-60%) in rural communities of Venezuela [23] and Ethiopia [50]. In case-control studies they have been more frequently identified in sick as compared to healthy children [44, 51, 60]. In this context, it is remarkable that these viruses were present in up to 72% of the infant samples we studied and in the absence of gastrointestinal symptoms. Like anelloviruses, caliciviruses were significantly more abundant in the second semester as compared to the first six months of life. The virus species most identified were Norwalk and Sapporo viruses, found in 70% and 20% of the samples, respectively, and they showed a high level of genetic diversity. We were able to assemble seven complete Norwalk virus genomes and they belonged to genotypes GI and GII; we also assembled two Sapporo virus genomes which belonged to the GII genotype. A more detailed description of the genetic variability of these viruses will be described elsewhere (Rivera-Gutiérrez et al., in preparation).

We identified a large set of 27 species of *Parvoviridae* and *Genomoviridae*, with most of them being insect and animal viruses. They were sporadically identified and in low abundance, possibly reflecting environmental contamination, except for human bocaviruses, which were identified in 11 of 35 infant samples. Such high prevalence was not surprising, as bocaviruses have been previously reported in feces of more than 40% of asymptomatic children [61]. Rotavirus A, a common etiological agent of infantile gastroenteritis, belonging to the *Reoviridae* family, was identified in 66% of the infant samples. Interestingly, all rotavirus reads detected showed an identity of 100% with different genes of the RotaTeq vaccine strains. The rotavirus vaccine was administered to the three infants at around two, forth, and six months of age, except for infant 5 who did not receive the last dose. Surprisingly, we identified the rotavirus vaccine strain in 4 out of 5 samples just before their first vaccination, which suggests a frequent transmission of the vaccine strain by close contact with vaccinated people, as it has been suggested in a previous study of transmission from vaccinated infants to their unvaccinated co-twin [62]. Rotavirus A was more abundant in the second semester (p-value 0.001, S6 Table), when the three doses had already been administered to infants.

Regarding plant-infecting viruses, those in the *Virgaviridae* family were frequently detected in the three children along the year of study. The *Tobamovirus* genus was the most frequent, with tropical soda apple mosaic virus, pepper mild mottle virus, and opuntia tobamovirus 2 being the most common species. Our results showed a large diversity of tobamoviruses circulating in the population, suggesting that infants are continuously exposed to an extensive and dynamic collection of these plant viruses, even before infants begin to ingest food other than mother’s breastmilk, including baby formula or other liquids, indicating a distinct source of origin for these viruses. We recently reported the genetic diversity and dynamics of tobamovirus infection in infants, as wells as the potential implications of these findings [42].

The richness of eukaryotic virus species found in this work was higher than in previous studies in healthy children, in which less than 9 different virus species were found per individual samples [13, 16, 23, 50, 51]. We identified on average 15 (±9 s.d.) virus species per sample, which is higher even when compared to previous reports in rural or small village communities [23, 50, 51]. In general, we also identified a greater number of enteric viruses compared to previous studies carried out not only in healthy infants, but also in sick children [44, 51]. Several factors may influence these results and should be considered in future studies. These include, an unbiased nucleic acid extraction method, which does not target only DNA viruses; depth of the sequencing carried out; socio-economic or demographic characteristics of the community or even a greater susceptibility or exposure of infants in our community as compared to other populations. When virus diversity in our samples was analyzed, it was found that the eukaryotic virome significantly increased in richness and abundance during the second semester of life, suggesting eukaryotic viruses are established as result of an increased environmental exposure of infants with age, in agreement with previous observations [16]. In line with this observation, viruses in the *Anelloviridae*, *Caliciviridae*, *Reoviridae* and *Virgaviridae* families were found more abundantly in the second semester, as compared to the first six months of life. It is important to mention that we estimated diversity based on annotated taxa, not at contig level, since in our experimental procedure virus-like particles were not purified; and thus, our values cannot be compared with those of previous studies that use this method.

Most viral reads in this work were assigned to bacteriophages, both in infants (84.5 ∓ 24%) and mothers (61.4 ∓ 37%). Unlike eukaryotic viruses, and in contrast to previous findings [16], no difference in richness was found between the first and second semester of life, although the mean abundance was greater in the first semester. Of note, bacteriophages were significantly richer and more diverse in infants than in their mothers, which agrees with a previous study where the richness and diversity of bacteriophages in the infant gut virome are reported to be higher than in adults, and decreases with age [14]. The dominant phages belonged to the *Siphoviridae*, *Myoviridae* and *Podoviridae* families in the *Caudovirales* order. Although previous studies have reported that the majority of gut bacteriophages seem to engage in lysogenic interactions with their hosts [6, 9, 63, 64], in our study the particular dominant phages in all samples were the recently described CrAssphages, an expansive diverse group of lytic bacteriophages with podovirus-like morphology that includes the most abundant viruses from the human gut [65]. In this regard, it is important to point out that these viruses have been reported to stably infect bacteria within the phylum Bacteroidetes during long periods of time both, *in vitro* and *in vivo* [47], although the mechanisms underlying this unusual relationship of carrier state-type are unknown. In any case, this type of interaction may start early in life, at least in the studied community.

CrAssphages have been reported to represent up to 95% of the total viral load in the adult´s gut, and to be present in 73% to 77% of samples analyzed in diverse human populations [65, 66]. Recent studies have shown that these viruses can be found as early as one week after birth [67] and it has been suggested that they could be vertically transmitted from mother to child [68]. In our study these phages were detected in 86% of the infants samples, with abundances ranging between 1% and 82% and with up to 96% of abundance in mothers; this frequency of detection was higher than that found in previous studies in infants, where they were detected in up to 53% of the samples [66]. More data is needed to understand the roles and dynamics of CrAssphage in gut equilibrium. Finally, functional analysis (S1 Fig), allowed to abundantly identify proteins associated with lysogenic viruses such as integrases and proteases, but also portal proteins which are used by lytic phages to form a pore that enables DNA passage during their packaging and ejection and endolysins that are used to degrade the cell wall from within, enabling viral progeny to be released. These results suggest that there is a core of virulent bacteriophages in early life of humans as there is in healthy adults [69].

The results and conclusions of this study are limited by a small sample size, although our primary goal was to have a first glimpse on the composition and dynamics of the eukaryotic and prokaryotic virome of healthy Mexican infants in a rural community during first year of life. Our findings suggest the existence of an early and constant contact of infants with a diverse array of eukaryotic viruses, whose composition changes over time. In addition to the phageome, that seems to be well established given the ubiquitous presence of their microbial hosts, the eukaryotic virus array could represent a metastable virome, whose potential influence on the development of the infant’s immune system or on the health of the infants during childhood, remains to be investigated.

## Supporting information

S1 Fig General metagenomic data analysis pipeline. * Minimally non-redundant nucleotide database from NCBI [31]. **Minimally non-redundant protein database from NCBI. ***Five million were the media of valid reads of all samples. + Threshold used to count as taxon.
(TIF)

S2 Fig Normalized reads abundance of eukaryotic viral families in infants during the first year of life, and their mothers. Samples from infants are indicated according to their age in months and the single mother sample in each case, as M. The virus family name is indicated on the left side, while the host and type of virus genome are shown in the right side. The discontinuous yellow lines divide the years into two semesters.
(TIF)

S3 Fig Normalized read abundance of eukaryotic viral genera in infants during the first year of life and their mother. Samples from infants are indicated according to their age in months and, from mothers, as M. The name of the viral genera is indicated on the left side while the family names are shown on the right side. The discontinuous yellow lines divide the year into semesters.
(TIF)

S4 Fig Normalized read abundance of bacteriophages families in infants during the first year of life and their mothers. Samples from infants are indicated according to their age in months and from mothers as M. The type of genome of each viral family is indicated on the right side.
(TIF)

S1 Table Characteristics of mother-infant binomials. (XLSX)

S2 Table Sampling summary and sequencing data generated for each sample. (XLSX)

S3 Table Reads abundance of eukaryotic viral species. (XLSX)

S4 Table Reads abundance of prokaryotic viral genera. (XLSX)

S5 Table Reads abundance of prokaryotic viral species. (XLSX)

S6 Table Taxonomic differential abundance between groups at different levels. (XLSX)
