# Supplementary figures and images for: The gut virome of healthy children during the first year of life is diverse and dynamic
Source: PLoS One. 2021 Apr 14;16(4):e0240958. doi: 10.1371/journal.pone.0240958 (PMC8046192; doi:10.1371/journal.pone.0240958)

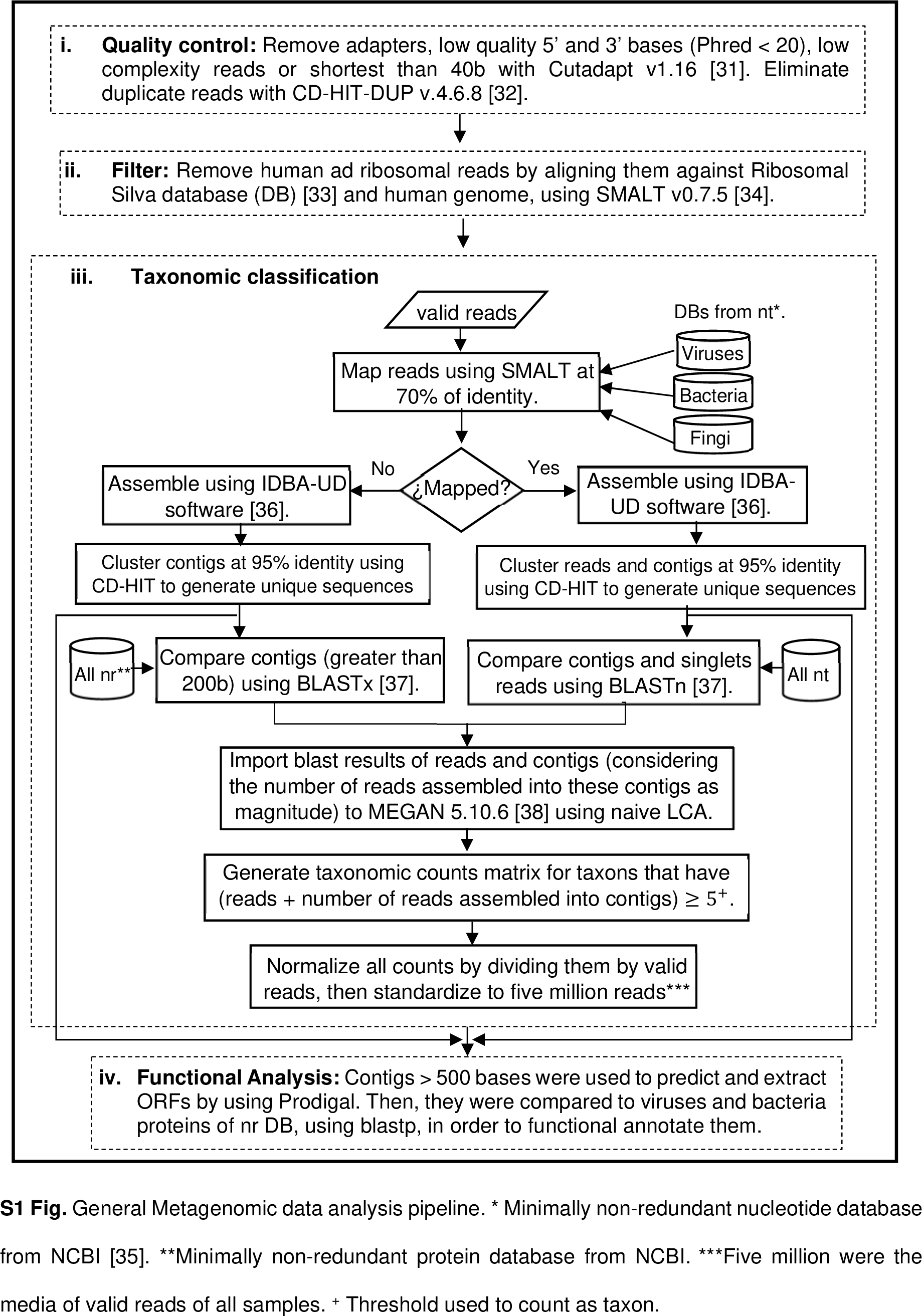

Supplement: S1 Fig — * Minimally non-redundant nucleotide database from NCBI [31]. **Minimally non-redundant protein database from NCBI. ***Five million were the media of valid reads of all samples. + Threshold used to count as taxon. (TIF) [file pone.0240958.s001.tif]

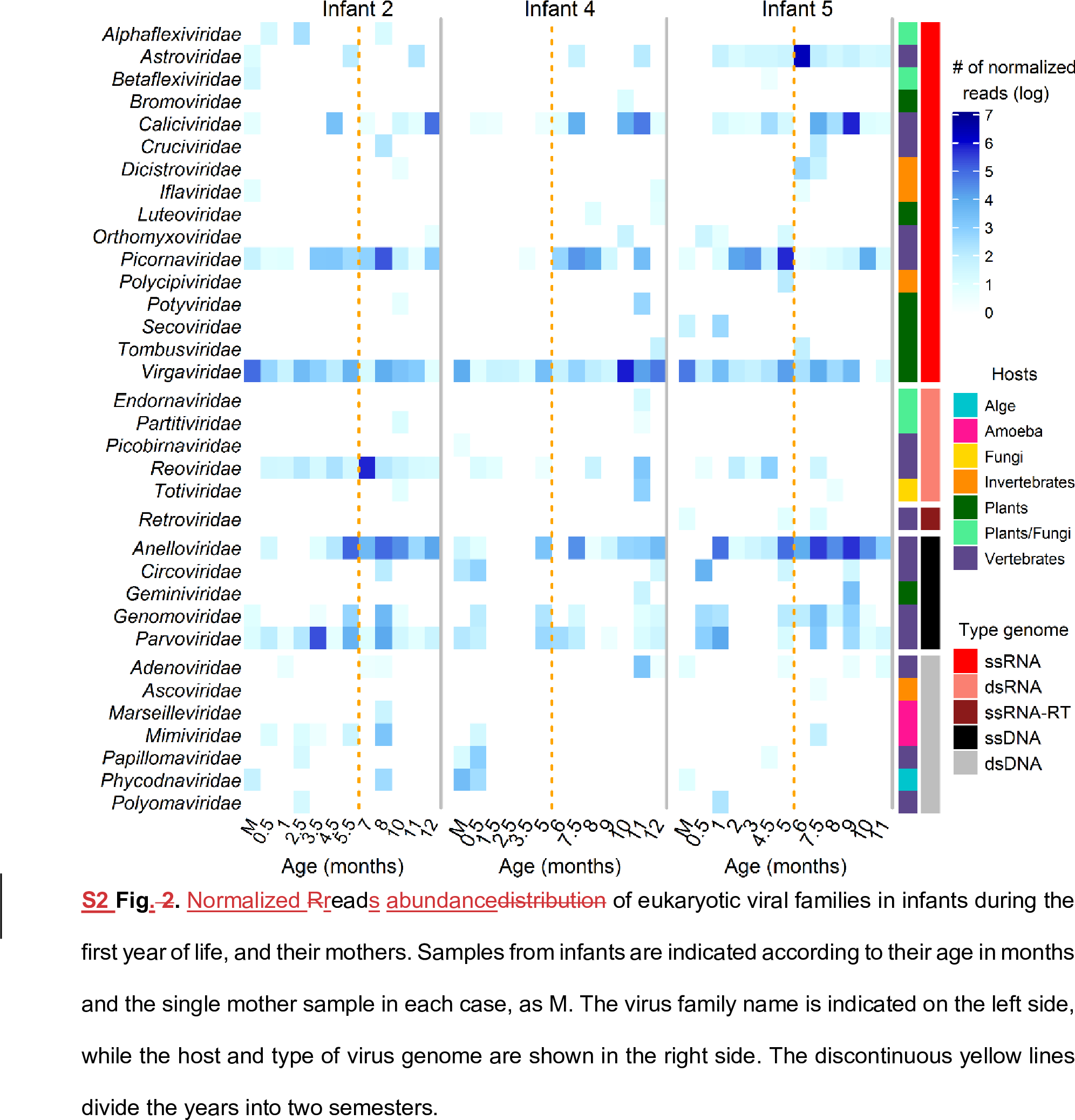

Supplement: S2 Fig — Samples from infants are indicated according to their age in months and the single mother sample in each case, as M. The virus family name is indicated on the left side, while the host and type of virus genome are shown in the right side. The discontinuous yellow lines divide the years into two semesters. (TIF) [file pone.0240958.s002.tif]

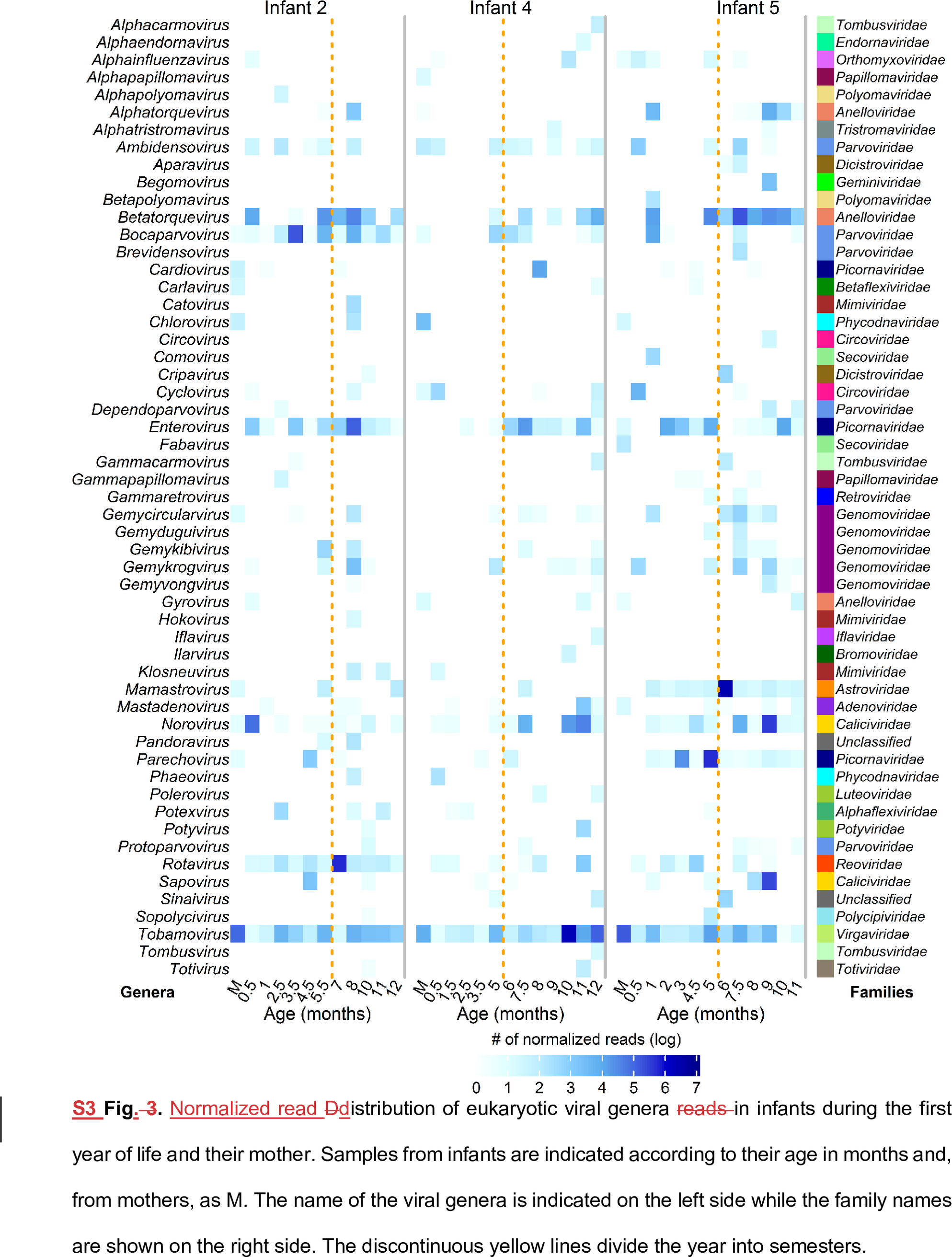

Supplement: S3 Fig — Samples from infants are indicated according to their age in months and, from mothers, as M. The name of the viral genera is indicated on the left side while the family names are shown on the right side. The discontinuous yellow lines divide the year into semesters. (TIF) [file pone.0240958.s003.tif]

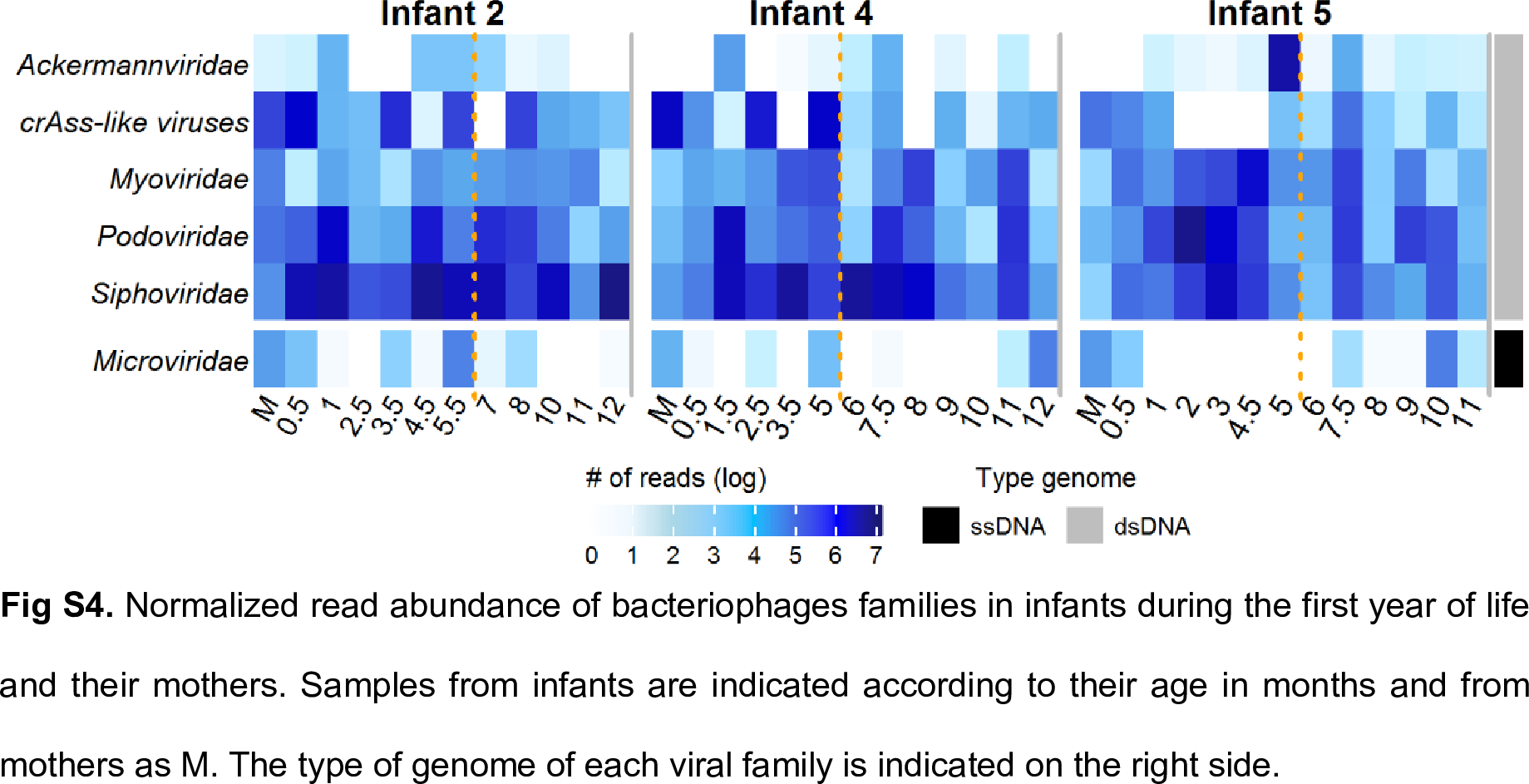

Supplement: S4 Fig — Samples from infants are indicated according to their age in months and from mothers as M. The type of genome of each viral family is indicated on the right side. (TIF) [file pone.0240958.s004.tif]
